# Supplementary material for: Multi‐dataset identification of innovative feature genes and molecular mechanisms in keratoconus
Source: J Cell Mol Med. 2024 Sep 19;28(18):e70079. doi: 10.1111/jcmm.70079 (PMC11412914; doi:10.1111/jcmm.70079)
Supplement: Supplementary file 2 — Material S2. [file JCMM-28-e70079-s002.zip › 7.GSEA analysis/1.ARL11/KEGG_NUCLEOTIDE_EXCISION_REPAIR.html]

Details for gene set KEGG\_NUCLEOTIDE\_EXCISION\_REPAIR[GSEA]

|  || Dataset | ARL11.group.cls#h\_versus\_l |
| Phenotype | group.cls#h\_versus\_l |
| Upregulated in class | h |
| GeneSet | KEGG\_NUCLEOTIDE\_EXCISION\_REPAIR |
| Enrichment Score (ES) | 0.24992794 |
| Normalized Enrichment Score (NES) | 1.084038 |
| Nominal p-value | 0.33526012 |
| FDR q-value | 1.0 |
| FWER p-Value | 0.998 |
Table: GSEA Results Summary

  

Fig 1: Enrichment plot: KEGG\_NUCLEOTIDE\_EXCISION\_REPAIR      
 Profile of the Running ES Score & Positions of GeneSet Members on the Rank Ordered List

  

| SYMBOL | TITLE | RANK IN GENE LIST | RANK METRIC SCORE | RUNNING ES | CORE ENRICHMENT || 1 | POLE2 | na | 3717 | 0.211 | -0.0236 | Yes |
| 2 | XPA | na | 4827 | 0.171 | 0.0121 | Yes |
| 3 | RPA4 | na | 5183 | 0.160 | 0.0653 | Yes |
| 4 | RFC3 | na | 7177 | 0.108 | 0.0506 | Yes |
| 5 | POLE | na | 8165 | 0.089 | 0.0573 | Yes |
| 6 | DDB1 | na | 8232 | 0.088 | 0.0901 | Yes |
| 7 | DDB2 | na | 8819 | 0.078 | 0.1039 | Yes |
| 8 | ERCC5 | na | 9044 | 0.074 | 0.1267 | Yes |
| 9 | ERCC8 | na | 9202 | 0.072 | 0.1506 | Yes |
| 10 | RPA1 | na | 10886 | 0.049 | 0.1214 | Yes |
| 11 | POLD1 | na | 11636 | 0.040 | 0.1156 | Yes |
| 12 | PCNA | na | 11742 | 0.039 | 0.1279 | Yes |
| 13 | ERCC4 | na | 11972 | 0.036 | 0.1358 | Yes |
| 14 | GTF2H5 | na | 13754 | 0.021 | 0.0927 | Yes |
| 15 | RBX1 | na | 14278 | 0.017 | 0.0842 | Yes |
| 16 | RAD23A | na | 15746 | 0.005 | 0.0441 | Yes |
| 17 | POLD4 | na | 16051 | 0.003 | 0.0367 | Yes |
| 18 | LIG1 | na | 16850 | -0.002 | 0.0146 | Yes |
| 19 | XPC | na | 17573 | -0.007 | -0.0034 | Yes |
| 20 | RPA2 | na | 18069 | -0.011 | -0.0134 | Yes |
| 21 | POLD3 | na | 18840 | -0.016 | -0.0292 | Yes |
| 22 | CUL4A | na | 18922 | -0.016 | -0.0251 | Yes |
| 23 | ERCC6 | na | 20343 | -0.026 | -0.0556 | Yes |
| 24 | GTF2H1 | na | 20649 | -0.029 | -0.0531 | Yes |
| 25 | POLE3 | na | 20917 | -0.031 | -0.0487 | Yes |
| 26 | CUL4B | na | 21172 | -0.033 | -0.0431 | Yes |
| 27 | CCNH | na | 21542 | -0.036 | -0.0397 | Yes |
| 28 | POLE4 | na | 22854 | -0.049 | -0.0582 | Yes |
| 29 | ERCC1 | na | 22905 | -0.049 | -0.0403 | Yes |
| 30 | GTF2H3 | na | 23197 | -0.052 | -0.0280 | Yes |
| 31 | ERCC3 | na | 23280 | -0.053 | -0.0093 | Yes |
| 32 | RAD23B | na | 23438 | -0.055 | 0.0079 | Yes |
| 33 | RFC5 | na | 23651 | -0.058 | 0.0247 | Yes |
| 34 | RPA3 | na | 23802 | -0.060 | 0.0440 | Yes |
| 35 | MNAT1 | na | 23860 | -0.060 | 0.0663 | Yes |
| 36 | POLD2 | na | 24003 | -0.062 | 0.0868 | Yes |
| 37 | ERCC2 | na | 24464 | -0.069 | 0.1008 | Yes |
| 38 | CETN2 | na | 24909 | -0.075 | 0.1176 | Yes |
| 39 | GTF2H4 | na | 24926 | -0.075 | 0.1469 | Yes |
| 40 | RFC4 | na | 25422 | -0.082 | 0.1649 | Yes |
| 41 | RFC2 | na | 25466 | -0.082 | 0.1963 | Yes |
| 42 | RFC1 | na | 25828 | -0.088 | 0.2205 | Yes |
| 43 | CDK7 | na | 26059 | -0.091 | 0.2499 | Yes |
Table: GSEA details [plain text format]

  

Fig 2: KEGG\_NUCLEOTIDE\_EXCISION\_REPAIR      
 Blue-Pink O' Gram in the Space of the Analyzed GeneSet

  

Fig 3: KEGG\_NUCLEOTIDE\_EXCISION\_REPAIR: Random ES distribution      
 Gene set null distribution of ES for **KEGG\_NUCLEOTIDE\_EXCISION\_REPAIR**
